# Characterization of response to atezolizumab + bevacizumab versus sorafenib for hepatocellular carcinoma: Results from the IMbrave150 trial

**DOI:** 10.1002/cam4.4090

**Published:** 2021-06-29

**Authors:** Riad Salem, Daneng Li, Nicolas Sommer, Sairy Hernandez, Wendy Verret, Beiying Ding, Riccardo Lencioni

**Affiliations:** ^1^ Department of Radiology Feinberg School of Medicine Northwestern University Evanston IL USA; ^2^ Department of Medical Oncology City of Hope Comprehensive Cancer Center and Beckman Research Institute Duarte CA USA; ^3^ Genentech, Inc South San Francisco CA USA; ^4^ Department of Radiology University of Pisa School of Medicine Pisa Italy; ^5^ Miami Cancer Institute Miami FL USA

**Keywords:** hepatocellular carcinoma, immunotherapy, Response Evaluation Criteria in Solid Tumors

## Abstract

**Background:**

IMbrave150 is a phase III trial that assessed atezolizumab + bevacizumab (ATEZO/BEV) versus sorafenib (SOR) in patients with unresectable hepatocellular carcinoma (HCC) and demonstrated a significant improvement in clinical outcomes. Exploratory analyses characterized objective response rate (ORR), depth (DpR), and duration of response (DoR), and patients with a complete response (CR).

**Methods:**

Patients were randomized 2:1 to intravenous ATEZO (1200 mg) + BEV (15 mg/kg) every 3 weeks or oral SOR (400 mg) twice daily. Tumors were evaluated using Response Evaluation Criteria in Solid Tumors version 1.1 (RECIST 1.1) and HCC‐modified RECIST (mRECIST). ORR by prior treatment and largest baseline liver lesion size, DoR, time to response (TTR), and complete response (TTCR) were analyzed.

**Results:**

For both criteria, responses favored ATEZO/BEV versus SOR regardless of prior treatment and in patients with lesions ≥3 cm. Median TTR was 2.8 months per RECIST 1.1 (range: 1.2–12.3 months) and 2.8 months per mRECIST (range: 1.1–12.3 months) with ATEZO/BEV. Patients receiving ATEZO/BEV had a greater DpR, per both criteria, across baseline liver lesion sizes. Characteristics of complete responders were similar to those of the intent‐to‐treat population. In complete responders receiving ATEZO/BEV per mRECIST versus RECIST 1.1, respectively, median TTCR was shorter (5.5 vs. 7.0 months), mean baseline sum of lesion diameter was longer (5.0 [SD, 5.1] vs. 2.6 [SD, 1.4] cm), and mean largest liver lesion size was larger (4.8 [SD, 4.2] vs. 2.3 [SD, 1.0] cm).

**Conclusions:**

These data highlight the improved ORR, DpR, and CR rates with ATEZO/BEV in unresectable HCC.

## Graphical abstract

IMbrave150 is a randomized phase III trial that showed statistically significant and clinically meaningful improvements in overall survival and progression‐free survival with atezolizumab plus bevacizumab versus sorafenib. The post hoc analyses presented here further highlight the ability of ATEZO/BEV to improve overall response rates, depth of response, and complete response rates in unresectable hepatocellular carcinoma versus sorafenib.

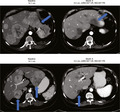

## 1 INTRODUCTION

Response Evaluation Criteria in Solid Tumors 1.1 (RECIST 1.1) is the established method for assessing the objective response rate (ORR).^1^, ^2^ It was developed for evaluating cytotoxic drugs, and it posits that tumor shrinkage is reflective of antitumor activity.^3^ Further delving into ORR, depth of response (DpR), a concept thoroughly described in colorectal cancer, aims to quantify the magnitude of response. DpR is poorly studied in hepatocellular carcinoma (HCC).^4^, ^5^, ^6^, ^7^

The use of RECIST 1.1 is controversial for assessing response to systemic therapy for HCC, given that these therapies mechanistically decrease tumor vascularity but may leave tumor size unaffected. The net effect is an underreporting of ORR.^8^ In addition, the assessment of progressive disease (PD) in HCC may be misinterpreted when relying solely on RECIST 1.1, due to imaging features associated with the natural history of cirrhosis (ascites, lymph node enlargement) that may be confused with PD.^9^ To account for the limitations of RECIST 1.1, HCC‐specific modified RECIST (mRECIST) was developed using contrast enhancement to identify viable lesions, incorporating vascular invasion, and explicitly defining patterns of progression, to ensure that PD is not inaccurately overreported.^8^, ^9^

IMbrave150 (NCT03434379) is a randomized phase III trial that assessed atezolizumab + bevacizumab (ATEZO/BEV) versus sorafenib (SOR) in patients with unresectable HCC who had not received prior systemic therapy.^10^ In the primary analysis (data cutoff: August 29, 2019), ATEZO/BEV resulted in a significant improvement in overall survival (OS) and progression‐free survival (PFS) compared with SOR, leading to regulatory approval and to becoming the new non‐tyrosine kinase inhibitor standard of care in this patient population.^10^, ^11^

Further, after 12 months of additional follow‐up, ATEZO/BEV maintained clinically meaningful survival benefits over SOR and a safety profile consistent with the primary analysis. In light of recent data on the antitumoral effects of immunotherapies,^12^, ^13^, ^14^, ^15^ we conducted further investigation with a post hoc analysis of the updated ORR, DpR, and complete response (CR) observed with immunotherapies in IMbrave150.

## 2 MATERIALS AND METHODS

### 2.1 Study design

Patients were randomly assigned (2:1) to receive intravenous ATEZO (1200 mg) (anti‐programmed death‐ligand 1; PD‐L1) + BEV (15 mg/kg) (anti‐VEGF) every 3 weeks or oral SOR (multikinase inhibitor) 400 mg twice daily until unacceptable toxicity or loss of clinical benefit.^10^ Eligibility criteria and randomization procedures have been described previously.^10^ Randomization stratification factors included geographic region (Asia excluding Japan vs. rest of world); macrovascular invasion (MVI), extrahepatic spread (EHS) of disease (presence vs. absence), or both; and baseline alpha‐fetoprotein level (<400 vs. ≥400 ng/ml).

Coprimary endpoints were OS and PFS by blinded independent central review (BICR)‐assessed RECIST 1.1. Key secondary endpoints included ORR and duration of response (DoR) per RECIST 1.1 and mRECIST.^8^ ORR by both response criteria was tested as part of a statistical hierarchy and was defined as the percentage of patients with a CR or partial response (PR). A confirmed objective response was defined as having been observed at two consecutive tumor assessments ≥28 days apart. DoR was defined as the time from first documented CR or PR to disease progression or death. Time to response (TTR) was defined as the time from randomization to the first confirmed response (either CR or PR) in all responders, and time to complete response (TTCR) was defined as the time from randomization to the first confirmed CR in all complete responders. DpR was defined as the percentage of tumor shrinkage based on longest diameter (LD), observed at the lowest point (nadir) compared with baseline. Tumors were assessed by computed tomography or magnetic resonance imaging at baseline and every 6 weeks until week 54, then every 9 weeks thereafter. Responses were analyzed by BICR with two independent radiologists and imaging adjudication by a third if there was a discrepancy.

Enrolled patients provided written informed consent. The study's protocol (available in the online supplement on the journal's website) was approved by each site's institutional review committee, and the study was conducted according to the ethical guidelines of the 1975 Declaration of Helsinki.

### 2.2 Current analyses

For ORR analyses, the ORs relative to SOR and the associated Wald 95% confidence intervals were estimated using logistic regression. ORR in the intent‐to‐treat (ITT) population with measurable disease at baseline was summarized by baseline characteristics and prior treatment, such as prior transarterial chemoembolization (TACE) (none, 1 or 2, or 3+ treatments) or baseline largest liver lesion (<3, ≥3 to <5, ≥5 to <7, or ≥7 cm).

DpR was determined by both minimum reduction (i.e., minimum percent change) in the sum of longest diameter (SLD) and the LD in the largest liver lesion for RECIST 1.1 and mRECIST in all patients and in subgroups according to baseline liver lesion size.

To better characterize patients who achieved a RECIST 1.1 or mRECIST CR, parameters such as baseline sum of target lesion; largest liver lesion size; number of prior TACE or doxorubicin‐eluting beads TACE (DEB‐TACE); Barcelona Clinic Liver Cancer (BCLC) stage at study entry; etiology; and presence of EHS, MVI, or both were descriptively analyzed. The full statistical analysis plan is available in the online supplement on the journal's website.

## 3 RESULTS

At the time of the clinical cutoff date for the updated analysis (August 31, 2020), the ORR was 30% (95% CI: 25%–35%) with ATEZO/BEV and 11% (95% CI: 7%–17%) with SOR per BICR RECIST 1.1, and 35% (95% CI: 30%–41%) and 14% (95% CI: 9%–20%), respectively, per mRECIST.^10^ Among responders (PR or CR), the median TTR with ATEZO/BEV was similar for both RECIST 1.1 and mRECIST (2.8 months; range: 1.2–12.3 months and 2.8 months; range: 1.1–12.3 months, respectively). In patients receiving SOR, the median TTR per mRECIST was shorter (2.6 months; range: 1.2–9.7 months) than per RECIST 1.1 (3.3 months; range: 1.2–5.7 months).

### 3.1 Objective response rate

**FIGURE 1:**
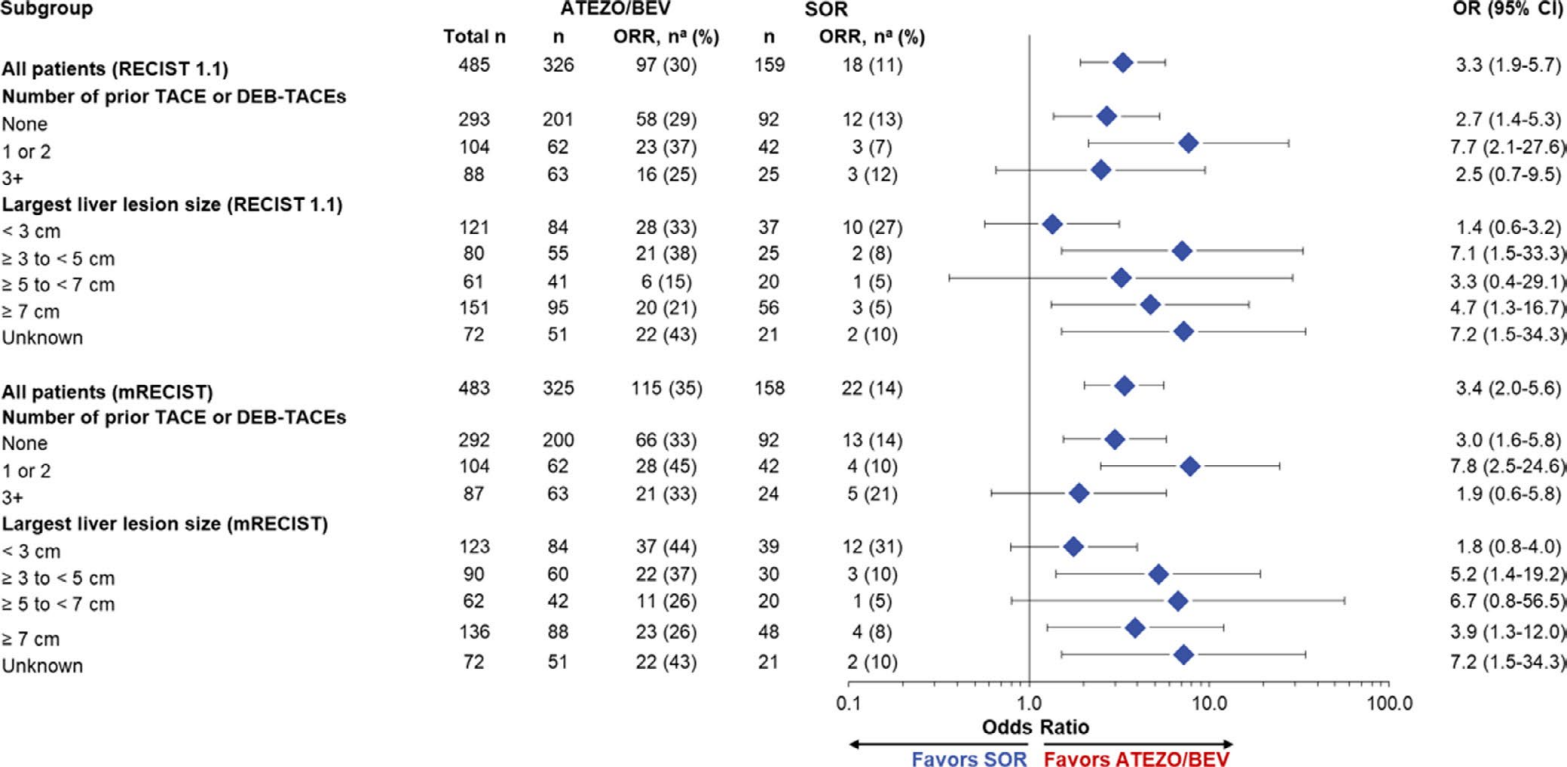
BICR‐assessed ORR per (RECIST 1.1 and mRECIST) in IMbrave150. ATEZO, atezolizumab; BEV, bevacizumab; BICR, blinded independent central review; CR, complete response; DEB‐TACE, doxorubicin‐eluting beads transarterial embolization; HCC, hepatocellular carcinoma; mRECIST, HCC‐modified Response Evaluation Criteria in Solid Tumors; ORR, objective response rate; PR, partial response; RECIST 1.1, Response Evaluation Criteria in Solid Tumors version 1.1; SOR, sorafenib; TACE, transarterial chemoembolization. ^a^Number of responders. Responders refer to all patients with CR or PR. A window of 28 days was used for the confirmation of CR or PR. OR relative to SOR and the associated Wald 95% CIs were estimated using unstratified logistic regression

To better understand the potential effect of tumor characteristics and prior locoregional therapy (LRT) when comparing ATEZO/BEV with SOR, we assessed ORR by largest lesion size and number of prior TACE/DEB‐TACEs (Figure 1). For this analysis, LRT refers to chemoembolization or chemoembolization with drug‐eluting beads (TACE or DEB‐TACE).

#### 3.1.1 Analysis by largest target liver lesion at baseline

Response in patients with largest target liver lesions ≥3 to <5, ≥5 to <7, or ≥7 cm favored ATEZO/BEV by both RECIST 1.1 (OR, 7.1 [95% CI: 1.5–33.3]; OR, 3.3 [95% CI: 0.4–29.1]; and OR, 4.7 [95% CI: 1.3–16.7], respectively) and mRECIST (OR, 5.2 [95% CI: 1.4–19.2]; OR, 6.7 [95% CI: 0.8–56.5]; OR, 3.9 [95% CI: 1.3–12.0], respectively). In patients who had lesions <3 cm, response also favored ATEZO/BEV per mRECIST (OR, 1.8 [95% CI: 0.8–4.0]).

#### 3.1.2 Analysis by prior LRT

Response rates were higher in the ATEZO/BEV arm than in the SOR arm, regardless of the number of prior LRTs for both BICR response criteria (Figure 1). The highest ORR was observed with ATEZO/BEV versus SOR in patients who received one or two prior LRTs (37.1% vs. 7.1% per RECIST 1.1; 45.2% vs. 9.5% per mRECIST).

### 3.2 Depth of response

#### 3.2.1 SLD % change

In the ITT population, patients receiving ATEZO/BEV had a greater DpR than those receiving SOR, regardless of BICR response criteria (Table 1). The mean (SD) minimum percentage SLD change was larger in the ATEZO/BEV arm than in the SOR arm per RECIST 1.1 (−27.3 [SD, 38.8] and −6.1 [SD, 29.4] in the ATEZO/BEV and SOR arms, respectively) and mRECIST (−38.1 [SD, 42.2] and −14.2 [SD, 36.7] in the ATEZO/BEV and SOR arms, respectively). A better DpR, as assessed by minimum percentage SLD changes, was observed across baseline liver lesion size, including larger tumors (≥7 cm; RECIST 1.1: −17.5 [SD, 29.3] and −4.5 [SD, 22.0] in the ATEZO/BEV and SOR arms, respectively; mRECIST: −27.9 [SD, 36.2] and −8.2 [SD, 28.0] in the ATEZO/BEV and SOR arms, respectively).

**TABLE 1:** Depth of responses in IMbrave150

	ITT population				Largest liver lesion ≥7 cm			
	RECIST 1.1		mRECIST		RECIST 1.1		mRECIST	
	ATEZO/BEV (*n* = 326)	SOR (*n* = 159)	ATEZO/BEV (*n* = 325)	SOR (*n* = 158)	ATEZO/BEV (*n* = 95)	SOR (*n* = 56)	ATEZO/BEV (*n* = 88)	SOR (*n* = 48)
Minimum percentage SLD change								
*n*	312	140	310	140	89	50	81	44
Mean (SD)	–27.3 (38.8)	–6.1 (29.4)	–38.1 (42.2)	–14.2 (36.7)	–17.5 (29.3)	–4.5 (22.0)	–27.9 (36.2)	–8.24 (28.0)
Median	–18.6	–2.3	–33.2	–6.7	–11.2	–0.4	–18.4	–4.1
Minimum percentage LD change in largest liver lesion								
*n*	262	120	255	119	89	50	81	44
Mean (SD)	–27.0 (36.0)	–7.4 (29.1)	–40.0 (42.5)	–18.5 (38.4)	–18.1 (29.6)	–5.8 (21.8)	–29.0 (37.1)	–9.5 (28.4)
Median	–20.2	–5.6	–30.9	–10.5	–11.2	–1.3	–18.4	–4.1

Abbreviations: ATEZO, atezolizumab; BEV, bevacizumab; ITT, intent‐to‐treat; LD, largest diameter; mRECIST, hepatocellular carcinoma‐modified Response Evaluation in Solid Tumors; RECIST 1.1, Response Evaluation Criteria in Solid Tumors version 1.1; SLD, sum of longest diameter; SOR, sorafenib.

#### 3.2.2 LD % change

DpR was also evaluated according to minimum percentage LD change in the largest liver lesion (Table 1; Table S1). Patients in the ITT population and the subgroups per baseline liver lesion sizes, including ≥7 cm, all exhibited similar trends, with a greater DpR across treatment arms and BICR response criteria. The mean (SD) minimum percentage LD change in the largest liver lesion was larger in the ATEZO/BEV arm than in the SOR arm per both response criteria (RECIST 1.1: −27.0 [SD, 36.0] and −7.4 [SD, 29.1] in the ATEZO/BEV and SOR arms, respectively; mRECIST: −40.0 [SD, 42.5] and −18.5 [SD, 38.4] in the ATEZO/BEV and SOR arms, respectively). In patients whose largest liver lesion was ≥7 cm, the mean minimum percentage LD changes in those lesions were also greater in the ATEZO/BEV arm than in the SOR arm per both RECIST 1.1 (−18.1 [SD, 29.6] and −5.8 [SD, 21.8] in the ATEZO/BEV and SOR arms, respectively) and mRECIST (−29.0 [SD, 37.1] and −9.5 [SD, 28.4] in the ATEZO/BEV and SOR arms, respectively).

### 3.3 Complete responders

#### 3.3.1 Overall analysis

By RECIST 1.1, 25 patients (8%) in the ATEZO/BEV arm achieved a CR; 1 patient (0.6%) achieved a CR in the SOR arm.^10^ By mRECIST, 39 patients (12%) receiving ATEZO/BEV and 4 patients (3%) receiving SOR achieved a CR.^10^ The median TTCR to ATEZO/BEV was 7.0 months (range, 1.2–18.8 months) per RECIST 1.1 and 5.5 months (range, 1.2–16.8 months) per mRECIST (Table 2). For both response criteria, the median DoR was not reached in patients receiving ATEZO/BEV who had CRs. One patient with CR (per RECIST 1.1 and mRECIST) had disease progression and one with CR (per mRECIST) died due to pneumonia. For patients who had CRs per mRECIST while receiving SOR, the median TTCR was 4.8 months (range, 1.4–9.2 months), and the median DoR had not been reached.

**TABLE 2:** Time to and duration of response in patients with a CR per BICR‐assessed RECIST 1.1 or mRECIST in IMbrave150

	RECIST 1.1		mRECIST	
	ATEZO/BEV (*n* = 25)	SOR (*n* = 1)	ATEZO/BEV (*n* = 39)	SOR (*n* = 4)
Median time to CR (range), months	7.03 (1.2–18.8)	9.2 (9.2–9.2)	5.5 (1.2–16.8)	4.8 (1.4–9.2)
Duration of response, months				
Patients without progression or death at cutoff, *n* (%)	18 (72)	1 (100)	27 (69)	2 (50)
Median (95% CI)	NE (14.5‐NE)	NE (NE‐NE)	NE (21.4‐NE)	17.9 (15.4–17.9)
Range	2.6+ to 22.4+	17.7+ to 17.7+	2.6+ to 23.6+	7.4+ to 17.9+
6‐months analysis				
Patients remaining at risk	22	1	35	4
Event‐free rate (95% CI), %	96 (88–100)	100 (100–100)	97 (92–100)	100 (100–100)

Abbreviations: +, censored; ATEZO, atezolizumab; BEV, bevacizumab; BICR, blinded independent central review; CI, confidence interval; CR, complete response; mRECIST, HCC‐modified Response Evaluation in Solid Tumors; NE, not evaluable; PR, partial response; RECIST 1.1, Response Evaluation Criteria in Solid Tumors version 1.1; SOR, sorafenib.

#### 3.3.2 Analysis by baseline characteristics

The characteristics of patients with a CR were generally similar to those of the ITT population per both response criteria, except that a smaller proportion (≥10% difference) of these patients had a history of alcohol use; MVI, EHS, or both; or no prior local therapy (Table 3). CRs per RECIST 1.1 and mRECIST were observed with ATEZO/BEV across all etiologies and baseline BCLC stage, regardless of the number of prior LRTs and tumor size. Patients receiving SOR who had a CR per mRECIST did not exhibit EHS or MVI at study entry.

**TABLE 3:** Baseline demographics and disease characteristics of patients with a CR per BICR‐assessed RECIST 1.1 or mRECIST in IMbrave150

Characteristics	ITT population	Patients with CR			
		RECIST 1.1		mRECIST	
	ATEZO/BEV (*N* = 336)	ATEZO/BEV (*n* = 25)	SOR (*n* = 1)	ATEZO/BEV (*n* = 39)	SOR (*n* = 4)
Median age (range), year	64 (26–88)	62 (37–84)	70 (70–70)	63 (37–87)	71 (62–78)
Age ≥65 year, *n* (%)	161 (48)	9 (36)	1 (100)	15 (39)	3 (75)
Men, *n* (%)	277 (82)	20 (80)	1 (100)	31 (80)	4 (100)
Race, *n* (%)					
Asian	188 (56)	16 (64)	0	28 (72)	1 (25)
White	123 (37)	9 (36)	1 (100)	11 (28)	3 (75)
Region, *n* (%)					
Asia (excluding Japan^a^)	133 (40)	13 (52)	0	24 (62)	0
Rest of world	203 (60)	12 (48)	1 (100)	15 (39)	4 (100)
Tobacco use history, *n* (%)					
Never	130 (38)	9 (36)	0	15 (39)	0
Current	54 (16)	3 (12)	0	5 (13)	2 (50)
Previous	152 (45)	13 (52)	1 (100)	19 (49)	2 (50)
Alcohol use history, *n* (%)					
Never	121 (36)	15 (60)	0	23 (59)	1 (25)
Current	48 (14)	1 (4)	0	2 (5)	1 (25)
Previous	166 (50)	9 (36)	1 (100)	14 (36)	2 (50)
ECOG PS at screening, *n* (%)					
0	209 (62)	19 (76)	1 (100)	28 (72)	3 (75)
1	127 (36)	6 (24)	0	11 (28)	1 (25)
Child‐Pugh score, *n* (%)					
A5	239 (72)	19 (76)	1 (100)	32 (82)	4 (100)
A6	94 (28)	6 (24)	0	7 (18)	0
APF ≥400 ng/ml, *n* (%)	126 (38)	6 (24)	1 (100)	12 (31)	3 (75)
Prior local therapy for HCC, *n* (%)	161 (48)	17 (68)	0	24 (62)	1 (25)
Prior TACE/DEB‐TACE, *n* (%)					
0	205 (61)	10 (40)	1 (100)	18 (46)	3 (75)
1 or 2	66 (20)	10 (40)	0	13 (33)	1 (25)
≥3	65 (19)	5 (20)	0	8 (21)	0
BCLC stage at study entry, *n* (%)					
Stage A1	8 (2)	2 (8)	0	2 (5)	0
Stage B	51(15)	5 (20)	0	8 (21)	1 (25)
Stage C	277 (82)	18 (72)	1 (100)	29 (74)	3 (75)
Etiology of HCC, *n* (%)					
HBV	164 (49)	15 (60)	0	24 (62)	1 (25)
HCV	72 (21)	6 (24)	0	9 (23)	0
Nonviral	100 (30)	4 (16)	1 (100)	6 (15)	3 (75)
MVI, *n* (%)^b^	129 (38)	7 (28)	1 (100)	15 (39)	1 (25)
EHS, *n* (%)^b^	212 (63)	14 (56)	1 (100)	22 (56)	1 (25)
MVI, EHS, or both, *n* (%)	258 (77)	16 (64)	1 (100)	27 (69)	1 (25)

Abbreviations: APF, alpha‐fetoprotein; ATEZO, atezolizumab; BCLC, Barcelona Clinic Liver Cancer; BEV, bevacizumab; BICR, blinded independent central review; CR, complete response; DEB‐TACE, doxorubicin‐eluting beads transarterial chemoembolization; ECOG PS, Eastern Cooperative Oncology Group performance status; EHS, extrahepatic spread; HBV, hepatitis B virus; HCC, hepatocellular carcinoma; HCV, hepatitis C virus; ITT, intent‐to‐treat; mRECIST, HCC‐modified Response Evaluation Criteria in Solid Tumors; MVI, microvascular invasion; RECIST 1.1, Response Evaluation Criteria in Solid Tumors version 1.1; SOR, sorafenib; TACE, transarterial chemoembolization.

^a^ Japan is included in the rest of the world.

^b^ At study entry, per electronic case report form.

#### 3.3.3 Analysis by largest target liver lesion size

The mean baseline SLD was lower in patients with CR receiving ATEZO/BEV than in the ITT population by both response criteria (RECIST 1.1: 2.6 [SD, 1.4] cm vs. 8.3 [SD, 6.0] cm, respectively; mRECIST, 5.0 [SD, 5.1] cm vs. 7.9 [SD, 5.6] cm, respectively; Table 4). The mean (SD) largest liver lesion size was also smaller in patients with CR than those in the ITT population per RECIST 1.1 and mRECIST. When comparing RECIST 1.1 and mRECIST, the mean baseline SLD in patients with CR receiving ATEZO/BEV was longer per mRECIST than per RECIST 1.1 (5.0 [SD, 5.1] cm vs. 2.6 [SD, 1.4] cm, respectively). Similarly, the mean largest liver lesion size in patients with CR receiving ATEZO/BEV was also larger per mRECIST than per RECIST 1.1 (4.8 [SD, 4.2] cm vs. 2.3 [SD, 1.0] cm, respectively). In patients with larger lesions (≥5 cm), CRs per mRECIST were observed only with ATEZO/BEV. Patients with CR per mRECIST receiving SOR had smaller mean baseline SLD and largest liver lesion size than patients receiving ATEZO/BEV (4.0 [SD, 1.0] cm vs. 5.0 [SD, 5.1] cm and 2.8 [SD, 1.8] cm vs. 4.8 [SD, 4.2] cm, respectively).

**TABLE 4:** Baseline tumor size by radiographic assessment in patients with a CR per BICR‐assessed RECIST 1.1 or mRECIST in IMbrave150

Characteristics	ITT population^a^					Patients with CR				
	RECIST 1.1			mRECIST		RECIST 1.1			mRECIST	
	ATEZO/BEV (*n* = 326)	SOR (*n* = 159)	ATEZO/BEV (*n* = 325)		SOR (*n* = 158)	ATEZO/BEV (*n* = 25)	SOR (*n* = 1)	ATEZO/BEV (*n* = 39)		SOR (*n* = 4)
Baseline sum of target lesion diameter (cm)										
Mean (SD)	8.3 (6.0)	8.8 (5.6)	7.9 (5.6)		8.3 (5.3)	2.6 (1.4)	4.7 (NE)	5.0 (5.1)		4.0 (1.0)
Median	6.9	7.9	6.5		7.3	2.0	4.7	2.7		4.1
Largest liver lesion size (cm)										
Mean (SD)	6.2 (4.4)^b^	6.6 (4.3)^c^	5.9 (4.2)^b^		6.3 (4.2)^c^	2.3 (1.0)^d^	NE (NE)^e^	4.8 (4.2)^f^		2.8 (1.8)^g^
Median	4.8^b^	5.5^d^	4.5^b^		4.9^c^	1.9^d^	NE^e^	2.8^f^		2.1^g^
Largest liver lesion size, *n* (%)										
<3 cm	84 (31)^b^	37 (27)^c^	84 (31)^b^		39 (29)^c^	12 (75)^d^	0^e^	16 (53)^f^		2 (67)^g^
≥3 to <5 cm	55 (20)^b^	25 (18)^c^	60 (22)^b^		30 (22)^c^	4 (25)^d^	0^e^	6 (20)^f^		1 (33)^g^
≥5 to <7 cm	41 (15)^b^	20 (15)^c^	42 (15)^b^		20 (15)^c^	0	0^e^	1 (3)^f^		0
≥7 cm	95 (35)^b^	56 (41)^c^	88 (32)^b^		48 (35)^c^	0	0^e^	7 (23)^f^		0

Abbreviations: ATEZO, atezolizumab; BEV, bevacizumab; BICR, blinded independent central review; CR, complete response; ITT, intent‐to‐treat; mRECIST, hepatocellular carcinoma‐modified Response Evaluation Criteria in Solid Tumors; RECIST 1.1, Response Evaluation Criteria in Solid Tumors version 1.1; SOR, sorafenib.

^a^ ITT patients with measurable disease at baseline.

^b^ *n* = 275 for RECIST 1.1 and *n* = 274 for mRECIST, respectively.

^c^ *n* = 138 for RECIST 1.1 and *n* = 137 for mRECIST, respectively.

^d^ *n* = 16.

^e^ *n* = 0.

^f^ *n* = 30.

^g^ *n* = 3.

**FIGURE 2:**
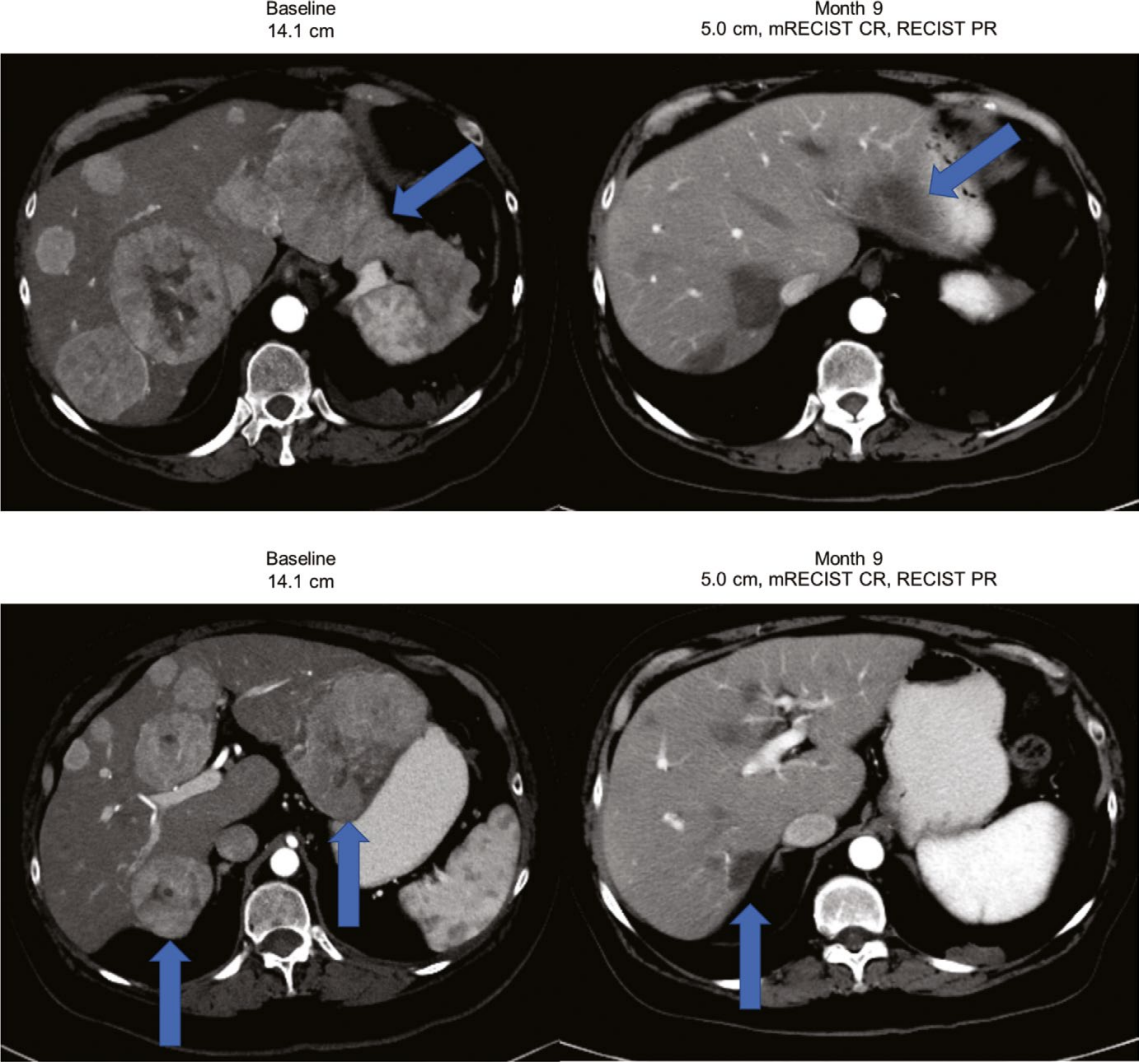
Example of a patient with HCC with multifocal bilobar disease that was highly vascularized. At baseline, the patient had multifocal bilobar disease that was highly vascularized, with a tumor that measured 14.1 cm. On the follow‐up scan 9 months later, the tumor had shrunk to 5.0 cm. The patient achieved a CR per mRECIST and a PR per RECIST 1.1. No prior locoregional therapy was reported, and treatment was still ongoing as of the date cutoff (August 2019) with a treatment duration of 294 days. CR, complete response; HCC, hepatocellular carcinoma; mRECIST, HCC‐modified Response Evaluation Criteria in Solid Tumors; PR, partial response; RECIST 1.1, Response Evaluation Criteria in Solid Tumors version 1.1

A case report of the difference between CR per mRECIST and PR per RECIST 1.1 is illustrated in Figure 2. Additional examples of patients who achieved a CR per mRECIST and a PR per RECIST 1.1 are shown in Figure S1.

## 4 DISCUSSION

Improving survival is one of the most important objectives of cancer treatment. Potential variables that could be surrogates are of interest and are currently under investigation. One proposed surrogate is ORR. This variable is one of the most commonly used parameters on which clinical decisions are based; knowing whether a tumor has responded to treatment by showing a decrease in size or necrosis is important for patients and physicians to decide whether to continue or discontinue therapy.^16^ Although most of the literature on ORR as a surrogate of OS has mainly focused on LRTs, there is growing interest in ORR in the literature about systemic therapies.^17^, ^18^ IMbrave150 showed statistically significant and clinically meaningful improvement in OS and PFS with ATEZO/BEV versus SOR, and several observations in ORR and DpR, as well as in which patients achieved CR, can be gleaned from this post hoc analysis.

IMbrave150 used both RECIST 1.1 and mRECIST methodologies to assess PFS (RECIST 1.1, primary endpoint) and response. A statistically significantly greater increase in ORR was observed with ATEZO/BEV versus SOR as assessed by BICR per RECIST 1.1 and mRECIST.^10^ The disease control rates were 73.6% and 55.3% with ATEZO/BEV and SOR, respectively. At the time of data cutoff for the primary analysis, responses were ongoing in 87% and 68% of responders in the ATEZO/BEV and SOR arms, respectively.^10^ Similar trends were observed using mRECIST. Per both response criteria, the median DoR had not yet been reached in the ATEZO/BEV arm versus 6.3 months in the SOR arm. The probability of having DoR ≥6 months was >80% in the ATEZO/BEV arm per both response criteria. This observation aligns with other studies, in which sustained and clinically relevant responses have been observed.^19^

The increase in ORR was maintained in the updated data. The ATEZO/BEV ORRs of 35% and 30% by mRECIST and RECIST are clinically meaningful and confirm the cytotoxic effect of the combination. Correlation between mRECIST and RECIST 1.1 was maintained for lesions up to 5 cm. However, expectedly, mRECIST responses appeared greater than RECIST once lesions exceeded 5 cm. This result is likely explained by avascular hepatic scarring, which can be observed in cirrhotic livers following treatment, simultaneously permitting mRECIST CR but preventing RECIST 1.1 CR (see Figure 2). Of interest, patients having received one or two LRTs appeared to have higher ORR than those receiving no or ≥3 LRTs. This result could be explained by a self‐selection bias, where patients never having received an embolization have unknown biological behavior, whereas those with ≥3 LRTs may have been treated excessively, without a natural transition from LRTs to systemic therapy. Hence, those who have received one or two treatments represent a self‐selected group and the ideal patient population, in whom biologic behavior is observed, liver functions are maintained, a natural transition to systemic therapy is made, and long‐term outcomes are optimized by allowing for sequential first‐ and second‐line drug therapies. A non‐systematic review has shown that some patients may benefit from early switch (i.e., after one or two TACE treatments) to systemic therapies rather than repeating treatment with LRT.^20^

As opposed to simply reporting the SLD when performing response assessments, analyzing the largest index lesion (i.e., target liver lesion) is of clinical importance.^21^ Large (≥7 cm) index lesions represent a significant clinical challenge and are assumed to be reflective of more aggressive tumor biology, lower response rates, and a tendency for LRT as first‐line options in liver‐only disease. Interestingly, the ATEZO/BEV combination generated a respectable 21% and 26% ORR by RECIST 1.1 and mRECIST, respectively, in patients with these challenging tumors, suggesting a role for systemic therapy in patients with larger lesions that are not candidates for, or have progressed with, LRTs. It further introduces the potential role of systemic agents in the downstaging setting, which is also space traditionally reserved for LRTs.^22^, ^23^, ^24^ With smaller tumors, the threshold for tumor reduction to meet response criteria is lower, which may explain the high response rate and longer PFS observed with SOR in lesions traditionally characterized as small (i.e., <3 cm).

In total, per RECIST 1.1, we observed a greater DpR with mean minimum percentage SLD changes of −27.3 (SD, 38.8) and −6.1 (SD, 29.4) in the ATEZO/BEV and SOR arms, respectively. The mean minimum percentage SLD change was −38.1 (SD, 42.2) and −14.2 (SD, 36.7) in the ATEZO/BEV and SOR arms, respectively, per mRECIST. These findings are clinically applicable, as they further reinforce the concept that although achieving a PR should be one goal of treatment, even minor reductions (not reaching PR threshold) in tumor size could translate to downstaging to ablation or transplant criteria. As an example, a patient with T3 disease and three lesions (2.5, 2.5, and 3.2 cm) could be downstaged to T2 transplant criteria with a 10% reduction of the 3.2 cm lesion, well within the observed DpR with ATEZO/BEV.^23^ This DpR was also maintained in larger index lesions (≥7 cm), with a mean minimum percent SLD change of −17.5% by RECIST 1.1 and −27.9% by mRECIST. The well‐described high ORR of cytotoxic LRTs for lesions ≥7 cm and the meaningful DpR and DoR with ATEZO/BEV suggest that these combinations represent the next natural evolution of clinical investigation.^16^

In a disease with historically low CR rates, it was notable that 25 (8%) and 39 (12%) patients achieved a CR per RECIST 1.1 and mRECIST, respectively, with ATEZO/BEV versus 0.6% and 3%, respectively, with SOR. The 6‐month event‐free rate was >90%, and the median DoR had not yet been reached. Furthermore, patients achieved CR across all etiologies and BCLC stage at entry, regardless of the number of prior LRTs and tumor size. Interestingly, complete tumor resolution was also seen in patients with MVI, an observation usually made only with LRTs such as Y90.^25^, ^26^ In patients demonstrating a prolonged CR, this observation is clinically meaningful and may confer a systemic therapy holiday (with strict imaging follow‐up). This concept is particularly relevant for patients who have limited options, in whom the preservation of future downstream treatment should be a strong consideration. In contrast, there were no mRECIST CRs in the SOR control arm in patients with baseline MVI or EHS, but the small number of patients achieving CR with SOR limits the interpretation of these data.

Recent guidance from an American Association for the Study of Liver Diseases panel of experts on clinical trial design recommended that the primary endpoint for systemic therapies should be OS, with PFS as a coprimary endpoint.^27^ For the assessment of ORR in response to systemic therapies, both RECIST 1.1 and mRECIST should be used.^27^ These recommendations are consistent with the design of the IMbrave150 study and the report herein.^10^

There are strengths and limitations to this analysis. Strengths include the source dataset being a large, multicenter, international phase 3 trial, with BICR using both RECIST 1.1 and mRECIST criteria. This is also the first analysis in HCC to describe the DpR and the potential clinical implications (e.g., downstaging), a concept usually reserved for colorectal cancer analyses. To further support the clinical relevance of these analyses, the data were stratified with granularity that permitted an exploratory look of patients with HCC managed in the real‐world setting. Limitations include the post hoc, retrospective analyses, with limited sample sizes in some subgroups. The presence of scarring identified at imaging following treatment may have resulted in the underreporting of CR and PR by RECIST 1.1, discrepancies with mRECIST, or both. There is a need for prospective investigations in bridging and downstaging groups, as well as combination LRT or systemic therapies. Furthermore, little is known about transplantation following immunotherapies; this field requires further study.

## 5 CONCLUSION

The data reported here describe the deeper ORR benefit that can be achieved with ATEZO/BEV than with SOR. Overall, responses achieved with ATEZO/BEV may be more clinically meaningful to patients with unresectable HCC and to clinicians who treat their patients with this combination as part of their standard of care. The findings also potentially introduce the concept of downstaging in patients who are ineligible for, or have had treatment failure with, LRTs. Further investigations in the neoadjuvant setting to resection, ablation, and transplant are warranted.

## CONFLICTS OF INTEREST

RS is a consultant for AstraZeneca, Boston Scientific, Cook, Eisai, Genentech, and Sirtex. DL has received fees for serving on a speakers bureau from Coherus Biosciences and Sun Pharmaceutical Industries; advisory board fees from Bayer Healthcare, Genentech, QED Therapeutics, and Taiho Pharmaceutical; fees for serving on a speakers bureau and advisory board from Advanced Accelerator Applications, Eisai, Exelixis, Ipsen Biopharmaceuticals, and Lexicon Pharmaceuticals; and a research grant to his institution from Brooklyn Immunotherapeutics. NS, SH, WV, and BD are employees and shareholders of Genentech, Inc. RL is a consultant for AstraZeneca, Eisai, and Genentech/Roche.

## Supporting information

Supplementary Material

## Data Availability

Qualified researchers may request access to individual patient‐level data through the clinical study data request platform at https://vivli.org/. Further details on Roche's criteria for eligible studies are available at: https://vivli.org/members/ourmembers/. For further details on Roche's Global Policy on the Sharing of Clinical Information and how to request access to related clinical study documents, see: https://www.roche.com/research_and_development/who_we_are_how_we_work/clinical_trials/our_commitment_to_data_sharing.htm.
