# Supplementary material for: Characterization of response to atezolizumab + bevacizumab versus sorafenib for hepatocellular carcinoma: Results from the IMbrave150 trial
Source: Cancer Med. 2021 Jun 29;10(16):5437–47. doi: 10.1002/cam4.4090 (PMC8366100; doi:10.1002/cam4.4090)

**SUPPLEMENT**

**SUPPLEMENTAL TABLE 1.** Depth of Responses in IMbrave150 Based on Largest Liver Lesion Size

|  | **<3 cm** | | | | **≥3 to <5 cm** | | | | **≥5 to <7 cm** | | | |
| --- | --- | --- | --- | --- | --- | --- | --- | --- | --- | --- | --- | --- |
|  | **RECIST 1.1** | | **mRECIST** | | **RECIST 1.1** | | **mRECIST** | | **RECIST 1.1** | | **mRECIST** | |
|  | **ATEZO/ BEV**  **(n=84)** | **SOR**  **(n=37)** | **ATEZO/ BEV**  **(n=84)** | **SOR**  **(n=39)** | **ATEZO/ BEV**  **(n=55)** | **SOR (n=25)** | **ATEZO/ BEV**  **(n=60)** | **SOR (n=30)** | **ATEZO/ BEV**  **(n=41)** | **SOR (n=20)** | **ATEZO/ BEV**  **(n=42)** | **SOR (n=20)** |
| **Minimum Percentage SLD Change** | | | | | | | | | | | | |
| n | 82 | 33 | 82 | 35 | 52 | 20 | 57 | 25 | 39 | 17 | 40 | 16 |
| Mean (SD) | −35.3 (41.0) | −16.9 (33.8) | −47.2 (43.1) | −33.7 (41.4) | −31.2 (35.8) | 0.8 (29.9) | −42.3 (40.3) | −9.4  (40.7) | −15.0(27.3) | 0.5 (29.7) | −33.8 (37.2) | −6.9 (29.9) |
| Median | −24.7 | −19.6 | −46.6 | −26.1 | −30.1 | −1.3 | −34.9 | −4.6 | −12.6 | −2.5 | −26.4 | −7.9 |
| **Minimum Percentage LD Change in Largest Liver Lesion** | | | | | | | | | | | | |
| n | 82 | 33 | 80 | 34 | 52 | 20 | 55 | 25 | 39 | 17 | 39 | 16 |
| Mean (SD) | −38.9 (40.8) | −15.8 (35.3) | −49.2 (46.2) | −36.6  (44.9) | −30.0 (38.9) | −5.0 (33.5) | −44.6  (43.3) | -17.2 (39.7) | −18.7  (26.7) | 1.0 (28.0) | −37.7 (39.7) | −6.7 (34.7) |
| Median | −32.1 | −14.0 | −47.0 | −38.7 | −28.8 | −11.2 | −37.9 | −13.7 | −18.3 | −4.7 | −26.7 | −8.7 |

ATEZO, atezolizumab; BEV, bevacizumab; LD, largest diameter; mRECIST; hepatocellular carcinoma-modified Response Evaluation Criteria in Solid Tumors; RECIST 1.1, Response Evaluation Criteria in Solid Tumors version 1.1; SLD, sum of longest diameter; SOR, sorafenib.

**Figure S1.** Additional examples. A, Patient with baseline vascular invasion into the hepatic vein. B, Patient with HCC with an infiltrative right lobe with portal vein thrombosis. CR, complete response; HCC, hepatocellular carcinoma; mRECIST, HCC-modified Response Evaluation Criteria in Solid Tumors; PR, partial response; RECIST 1.1, Response Evaluation Criteria in Solid Tumors version 1.1.


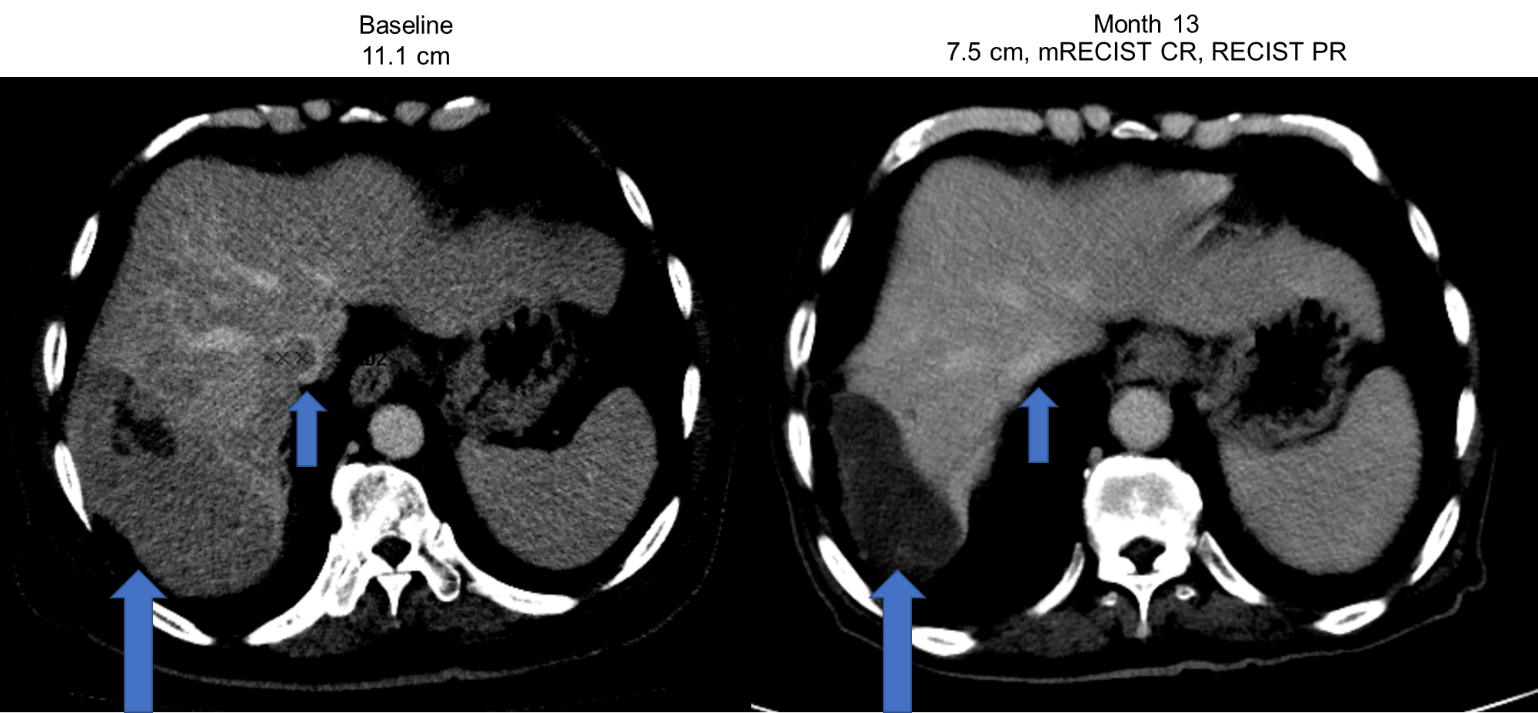


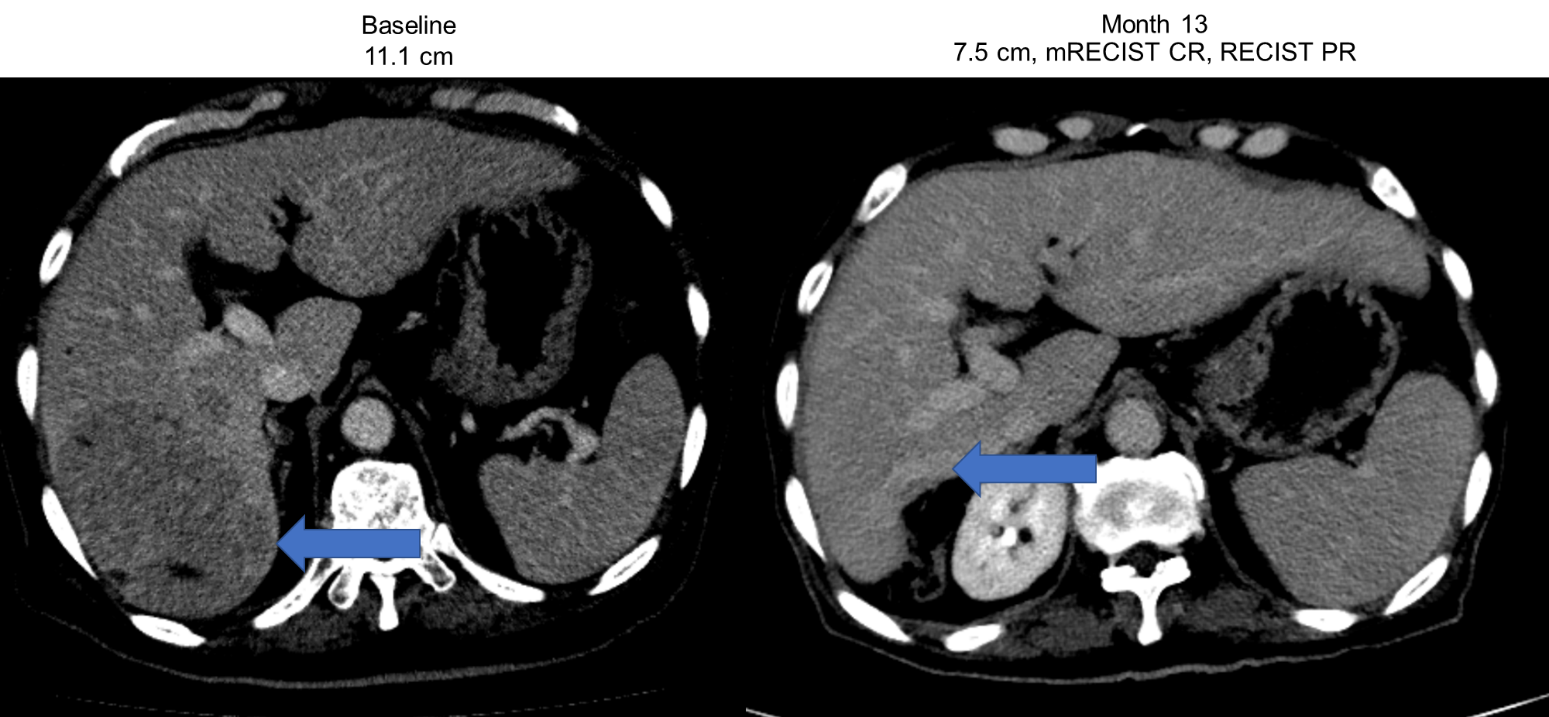


B.


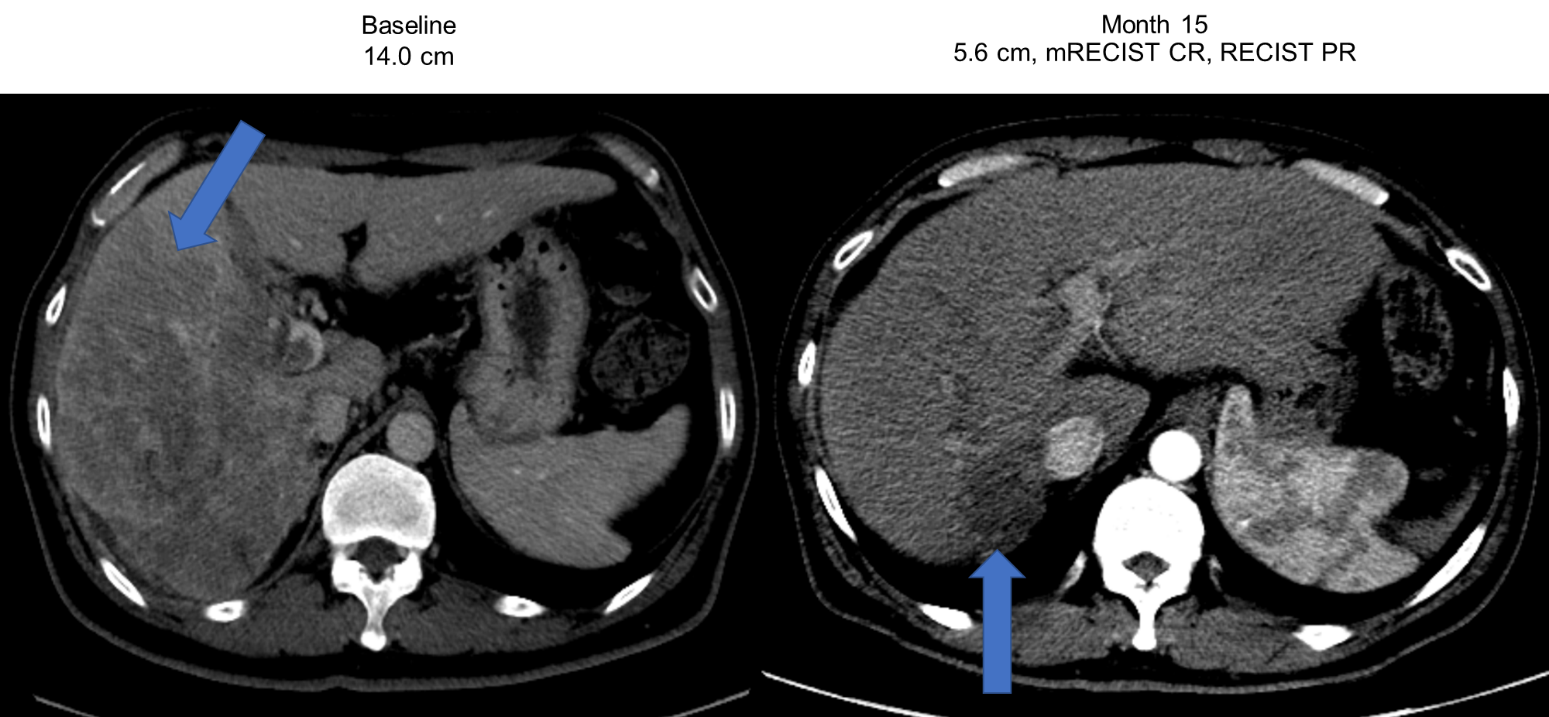


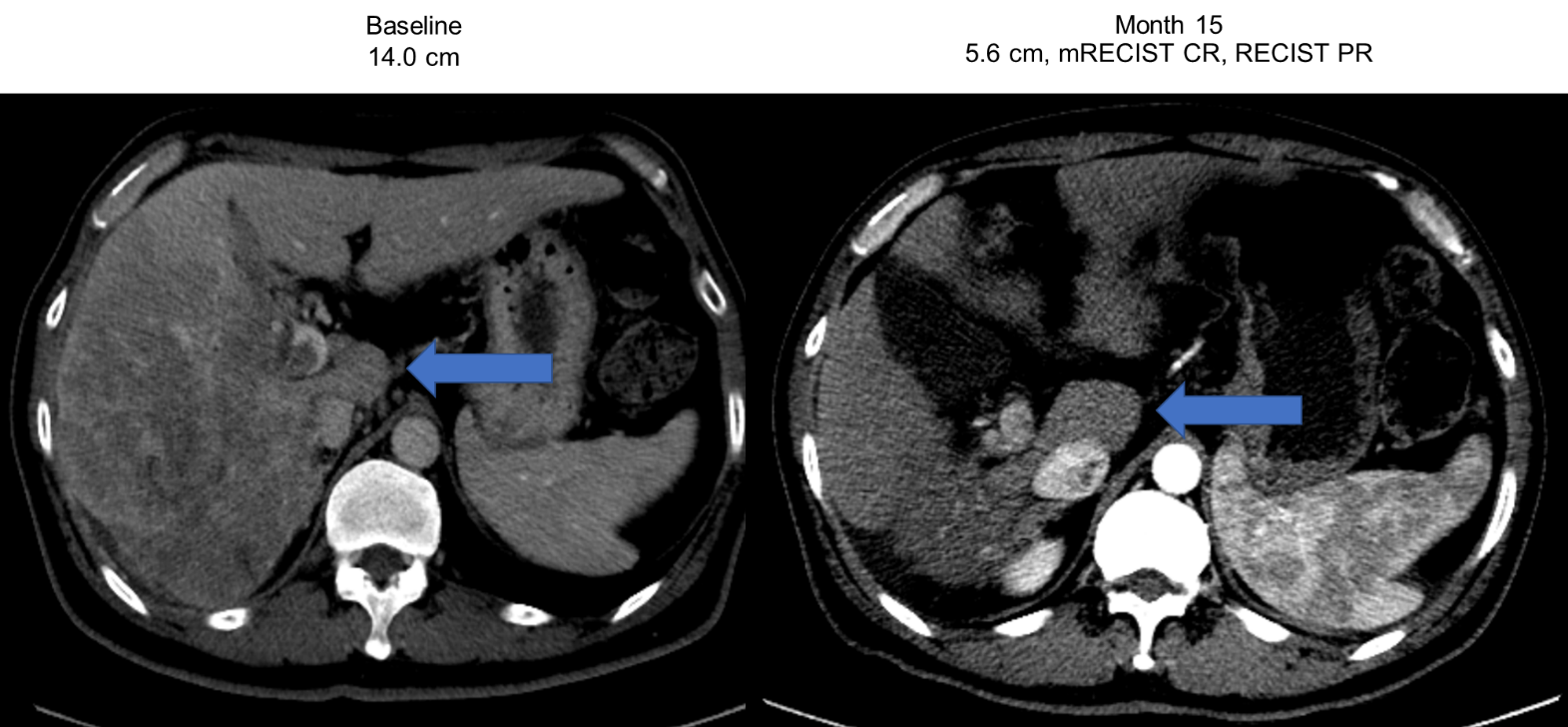

Supplement: Supplementary file 1 — Supplementary Material [file CAM4-10-5437-s001.docx]
